# Randomized trial on acute toxicities of weekly vs three‐weekly cisplatin‐based chemoradiation in head and neck cancer

**DOI:** 10.1002/cnr2.1425

**Published:** 2021-06-07

**Authors:** Ahmad Ameri, Shokoufe Norouzi, Ainaz Sourati, Samira Azghandi, Kambiz Novin, Farzad Taghizadeh‐Hesary

**Affiliations:** ^1^ Department of Clinical Oncology, Imam Hossein Educational Hospital Shahid Beheshti University of Medical Sciences Tehran Iran; ^2^ Department of Clinical Oncology, Shohada‐e Tajrish Educational Hospital Shahid Beheshti University of Medical Sciences Tehran Iran; ^3^ Department of Clinical Oncology, Faculty of Medicine Iran University of Medical Sciences Tehran Iran; ^4^ Department of Clinical Oncology Shahid Beheshti University of Medical Sciences Tehran Iran

**Keywords:** acute toxicity, chemoradiation, cisplatin, head and neck cancer, hematologic toxicity, renal toxicity

## Abstract

**Background:**

The current first‐line treatment of locally advanced head and neck carcinoma (LAHNC) is concurrent chemoradiation with three‐weekly cisplatin 100 mg/m^2^. However, prescribing cisplatin at this dose increases the treatment toxicity, which may compromise the treatment results. An alternative schedule is weekly 40 mg/m^2^ cisplatin.

**Aim:**

To compare the acute hematologic and renal toxicities of these two regimens.

**Methods:**

This randomized clinical trial included 77 LAHNC patients who were allocated to a high dose (100 mg/m^2^ every 3 weeks) or low dose (40 mg/m^2^ weekly) cisplatin group concurrent with radiotherapy. Hematologic and renal indices were measured weekly during chemoradiation.

**Results:**

The average age of patients was 55.3 years. Overall, 71.4% of patients were treated in a definitive setting. The incidence of severe hematologic events was not significantly different. However, the average estimated glomerular filtration rate (eGFR) was significantly greater in the three‐weekly group (67.85 vs. 58.57% mL/min per 1.73 m^2^; *P*‐value = .02). Cumulative cisplatin dose of ≥240 mg/m^2^ was significantly greater in the weekly group. Totally, treatment breaks occurred in 40.3% of patients due to treatment toxicity. Treatment interruption was primarily due to neutropenia in the three‐weekly and renal dysfunction and thrombocytopenia in the weekly group.

**Conclusions:**

Severe acute hematologic toxicities were comparable for three‐weekly and weekly groups. The decrease in eGFR through treatment was more significant with weekly cisplatin. Further follow‐up, however, is needed to confirm its impact on delayed renal function.

## 1 INTRODUCTION

Administration of chemotherapy concurrent with radiotherapy has improved significantly the prognosis of patients suffering from locally advanced head and neck carcinoma (LAHNC) not only by achieving an impressive response rate but also by improving overall survival. This statement is true for both definitive and postoperative settings.^1^, ^2^ On the other hand, chemoradiation induces more toxicities that eventually result in suboptimized treatment in about 40% of the patients.^3^, ^4^, ^5^ Concurrent cisplatin is currently the most widely used choice for LAHNC. According to the National Comprehensive Cancer Network guidelines for head and neck cancers (Version 3.2019), the recommended chemotherapy regimen concurrent with radiotherapy includes cisplatin 100 mg/m^2^ (in terms of response rate and survival) administered every 3 weeks for two or three cycles based on radiation fractionation scheme.^6^, ^7^ However, the three‐weekly regimen is frequently associated with adverse effects that may result in delay or interruption of chemotherapy, which then causes less cumulative doses of cisplatin.^8^ A retrospective review conducted in the United Kingdom revealed that all patients failed to receive the full three cycles of 100 mg/m^2^ cisplatin concurrent with radiotherapy in a definitive setting.^9^

Alternative dosing schedules of cisplatin were adopted to improve the compliance of patients. One of the most commonly used regimens administered along with radiotherapy is weekly cisplatin of 40 mg/m^2^.^3^, ^8^, ^9^, ^10^, ^11^, ^12^ Existing data comparing the toxicity profiles of weekly versus three‐weekly cisplatin are inconclusive.^3^, ^8^, ^10^, ^12^, ^13^ This prospective clinical trial aimed to compare the acute hematologic and renal toxicity profiles following weekly and three‐weekly dosing schedules of cisplatin to assist clinicians with their clinical decisions.

## 2 MATERIALS AND METHODS

### 2.1 Trial design and randomization

This study was a prospective, open‐label randomized trial of patients with LAHNC who were admitted to the Imam Hossein Educational Hospital, Tehran, Iran. The statistical team was blinded to the treatment process while the participants and researchers were not blinded (i.e., open‐label). The randomization was stratified according to the age, Karnofsky performance status, primary site, the setting of the treatment (i.e., definitive or postoperative), receiving/not receiving induction chemotherapy, and the type of the induction protocol (docetaxel plus cisplatin plus fluorouracil vs. paclitaxel plus carboplatin protocol). Within each stratum, the patients were randomly assigned to receiving cisplatin‐based concurrent chemoradiotherapy (CCRT) weekly (40 mg/m^2^) or three‐weekly (100 mg/m^2^). Thereafter, the permuted blocks technique was used for the randomization.

### 2.2 Participants

The inclusion criteria were: (1) previously untreated patients with clinical stage III–IV LAHNC [T3‐4, N0‐3, M0; based on the AJCC staging system (seventh edition)]; (2) patients aged at most 80 years and at least 20 years with an Eastern Cooperative Oncology Group (ECOG) of 0–2; (3) patients with adequate renal and bone marrow function (defined as the glomerular filtration rate of ≥60, an absolute neutrophil count of ≥1.5 × 10^3^/mL, hemoglobin level ≥ 10 g/dL, and platelet count ≥100 × 10^3^/mL). Patients with a synchronous or metachronous cancer, psychological disorder, uncontrolled underlying disease (e.g., diabetes mellitus, hypertension), or contraindications for treatment with platinum (including peripheral neuropathy, renal failure, and uncontrolled thrombocytopenia) were excluded. Patients who did not agree to stop smoking and/or drinking alcohol were also excluded from the study. Based on previous studies,^12^ with an error of 5% and a statistical power of 80%, 50 patients were required for each group.

### 2.3 Intervention and outcome

The initial assessment consisted of a complete history and physical examination, computed tomography (CT), and/or magnetic resonance imaging (MRI) for locoregional extension of disease (if patients were treated in definitive setting), chest X‐ray, abdominal ultrasonography, complete blood cell count (CBC), and comprehensive blood chemistries, including serum creatinine (Scr), electrolytes, and liver function tests. Clinical examination, CBC, and blood chemistry were repeated before each course. To evaluate renal function, we used three parameters: the marked rise in Scr (defined as Scr ≥3.0 × baseline), development of acute kidney injury (AKI; defined as Scr ≥3.0 × baseline, increase to ≥4.0 mg/dL, or initiation to renal replacement therapy),^14^ and marked drop in estimated glomerular filtration rate (eGFR; refer to Table S1).

**FIGURE 1:**
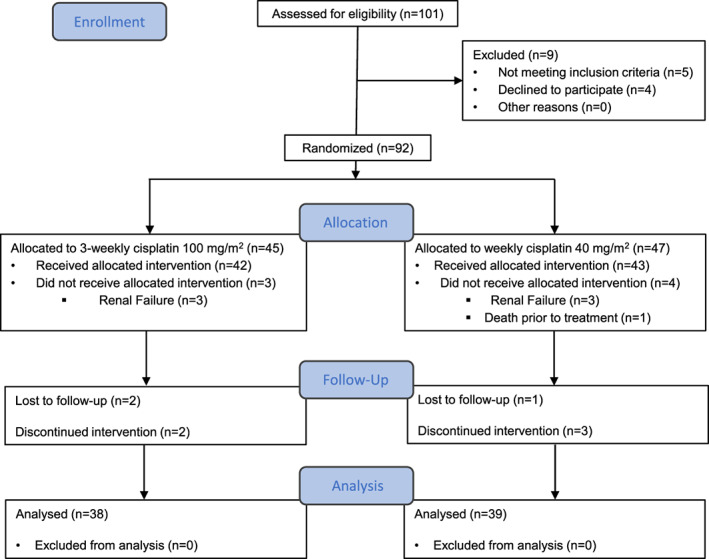
The CONSORT flow diagram

Figure 1 shows the consort diagram of the trial. Radiotherapy was delivered using the three‐dimensional conformal technique at a daily dose fraction of 2 Gy for 5 days per week. Patients were randomly assigned to receive cisplatin as either a weekly 40 mg/m^2^ regimen (AKA as the low dose group) or a three‐weekly 100 mg/m^2^ regimen (AKA as the high dose group), both concurrent with radiotherapy. In the weekly arm, participants were treated in the out‐patient setting with 1 L of 0.9% normal saline administered intravenously during 1 h as prehydration followed by another 1 L of 0.9% normal saline containing cisplatin, which was infused over an hour.

Patients in the three‐weekly schedule were treated in the in‐patient setting. Prehydration included an infusion of 1 L of 0.9% normal saline containing 10 cc potassium chloride (%15) and 2.5 g of 50% magnesium sulfate administered in 3 h followed by 100 cc mannitol 20% and 20 mg furosemide prescribed as an intravenous short infusion. The prescribed dose of cisplatin was given in 1 L of normal saline 0.9% for 4 h followed by 1 L of normal saline containing 10 cc potassium chloride and 2.5 g magnesium sulfate for 3 h as posthydration. Dose adjustments were based on blood counts and creatinine clearance that were taken prior to each cycle. Creatinine clearance was evaluated with the Cockcroft‐Gault formula (eGFR). Dose adjustment and delay in treatment were performed using British Columbia cancer (BC cancer) institute protocol^15^ (Tables S1 and S2).

### 2.4 Follow‐up

After the treatment, patients were assessed at 12 weeks and then every 3 months for the first 2 years.

### 2.5 Evaluation criteria

To evaluate and grade the treatment‐induced toxicities, the Common Terminology Criteria for Adverse Events (CTCAE) v4.03 was utilized.^16^ Dose‐limiting toxicity was defined as the occurrence of at least one of the following events: (1) renal toxicity in the form of eGFR less than 60 mg/mL; (2) hematological toxicity in the form of absolute neutrophil count (ANC) ≤800 mL^−1^ and/or platelet count ≤100 × 10^3^ mL^−1^; or (3) death.

### 2.6 Statistical analyses

The primary objective of this trial was to compare the rate of hematological and renal toxicities of three‐weekly cisplatin with weekly cisplatin—concurrent with radiotherapy—in patients with LAHNC. The data management and analysis were performed using SPSS v21.0 (IBM Corp., Armonk, NY) software. The Chi‐square test and Fischer's exact test were used to evaluate the difference between the control and experimental groups. Moreover, the Mann‐Whitney *U* test and independent *t*‐test were conducted to analyze continuous variables. The generalized estimating equation (GEE) was run to assess the patients' eGFR over the trial. The statistical significance level was set to .05.

## 3 RESULTS

We screened 101 participants with LAHNC for eligibility. Of these, 92 patients (91.1%) were randomly assigned to the study groups (Figure 1). The patients were followed up for at least 12 weeks. The final analysis included 39 and 38 patients who assigned to weekly and three‐weekly cisplatin regimens, respectively.

### 3.1 Patients' characteristics

Table 1 provides an overview of participants' characteristics. The mean age was 55.3 years (21–82 years), and 60 patients (77.9%) were male. Both groups were evenly balanced according to age, gender, smoking status, primary site, pathology subtype, and the clinical stage. The larynx was the most common primary site (49.3%) and squamous cell carcinoma (SCC) was the most common pathology (81.8%). Ten patients had undifferentiated carcinoma and two patients had adenoid cystic carcinoma. Furthermore, 22.1% and 75.3% of the participants had stage III and IV disease, respectively. In line with this, two patients with stage II and three metastatic cases were also enrolled in this study based on clinical discretion.

**TABLE 1:** Patients' demographic and clinical characteristics

Characteristics	Weekly (*n* = 39)	Three‐weekly (*n* = 38)	*P*‐value
Mean age (years)	55.46	55.24	.942
Standard deviation	± 13.30	±13.23	
Range	23–75	21–82	
Gender			.737
Female	20.5% (8)	23.7% (9)	
Male	79.5% (31)	76.3% (29)	
Smoking history			.712
Yes	58.9% (23)	52.8% (19)	
No	41.0% (16)	47.2% (17)	
Location of primary site			.935
Nasopharynx, nasal cavity, paranasal‐sinuses	15.4% (6)	18.4% (7)	
Oropharynx, oral cavity, salivary glands	28.2% (11)	26.3% (10)	
Larynx, hypopharynx	56.4% (22)	55.3% (21)	
Pathology			.335
SCC	84.6% (33)	78.9% (30)	
Non‐SCC	15.3% (6)	21.1% (8)	
T stage			.852
T_1_	2.6% (1)	2.6% (1)	
T_2_	17.9% (7)	10.5% (4)	
T_3_	35.9% (14)	36.8% (14)	
T_4_	43.6% (17)	50.0% (19)	
N stage			.731
N_0,1_	48.7% (19)	52.6% (20)	
N_2,3_	51.3% (20)	47.4% (18)	
M stage			.615
M_0_	97.4% (38)	94.7% (36)	
M_1_	2.6% (1)	5.3% (2)	
Clinical stage			.573
S II	5.1% (2)	–	
S III	23.1% (9)	21.1% (8)	
S IV	71.8% (28)	78.9% (30)	

Abbreviation: SCC, squamous cell carcinoma.

### 3.2 Treatment and evaluation details

The results obtained from the preliminary analysis of both treatment schedules are summarized in Table 2. In this trial, 55 patients received chemoradiation as definitive (28 and 27 patients in weekly and three‐weekly arms, respectively) and the remaining 22 patients received chemoradiation as an adjuvant treatment. The median survival of definitive and adjuvant groups were 29 and 32 months, respectively. In the definitive setting, 50 patients (90.9%) received induction chemotherapy. Six and eleven cases of the weekly and three‐weekly arms had received cisplatin‐based induction chemotherapy, respectively. Eighteen and fifteen cases in the weekly and three‐weekly arms had received carboplatin‐based induction chemotherapy, respectively. The mean cumulative dose of cisplatin in the induction regimen of weekly and three‐weekly schedules was 188.75 and 218.18 mg/m^2^, respectively. This difference was not statistically significant (*P*‐value = .53).

**TABLE 2:** The analysis of treatment status

Treatment status	Weekly (*n* = 39)	Three‐weekly (*n* = 38)	*P*‐value
Overall treatment time			.288
Mean (±SD)	50.54 (±7.29)	49.03 (±6.49)	
Range	51–76	39–65	
Mean radiation dose (Gy) (±SD)	64.95 (±4.23)	63.37 (±3.97)	.156
Cumulative dose of cisplatin (mg/m^2^)			.321
Average (±SD)	251.79 (±39.66)	239.87 (±50.12)	
Range	160–320	175–300	
Cumulative dose of cisplatin ≤200 mg/m^2^ ^a^	94.9% (37)	81.6% (31)	.087
Cumulative dose of cisplatin ≤240 mg/m^2^ ^a^	79.5% (31)	52.6% (20)	**.017**
Chemotherapy dose reduction	33.3% (13)	42.1% (16)	.427
Reasons for dose reduction			**.001**
Neutropenia	7.7% (1)	68.7% (11)	
Thrombocytopenia	92.3% (12)	6.3% (1)	
Renal disorders	–	25% (4)	
Rate of delay/cessation in chemotherapy	38.4% (15)	42.1% (16)	.707
Reasons for delay/cessation in chemotherapy			
Neutropenia	6.6% (1)	81.2% (13)^b^	**.001**
Thrombocytopenia	13.3% (2)	6.2% (1)	.999
Renal disorders	73.3% (11)	6.2% (1)	**.001**
Other reasons (e.g., mucositis)	6.6% (1)	25% (4)	.431
Radiation gap	10.2% (4)	15.7% (6)	.895
Reasons for radiation gap			
Neutropenia	–	66.6% (4)^c^	.194
Thrombocytopenia	75% (3)	16.6% (1)	.088
Mucositis	25% (1)	33.3% (2)	.667
Significant weight loss (≥10%)	43.6% (17)	42.1% (16)	.895

*Note: P*‐values less than 0.05 are significant.

^a^ The cut off values are extracted based on the study by Ho et al.^9^

^b^ Three patients experienced neutropenia in accordance with other toxicities.

^c^ One patient experienced neutropenia and thrombocytopenia.

### 3.3 Radiotherapy details

All patients completed the planned radiation course. The median chemoradiation duration was 50.54 days for the weekly arm and 49.03 days for the three‐weekly arm, respectively, without significant difference (*P*‐value = .28). Radiotherapy break occurred in four cases of weekly group (three cases due to thrombocytopenia and one case due to severe mucositis) and six cases of three‐weekly group (three cases due to neutropenia, one case due to neutropenia and thrombocytopenia, and two cases due to severe mucositis). The maximum duration of the treatment break was 8 and 11 days for the weekly and three‐weekly groups, respectively.

### 3.4 Chemotherapy details

**FIGURE 2:**
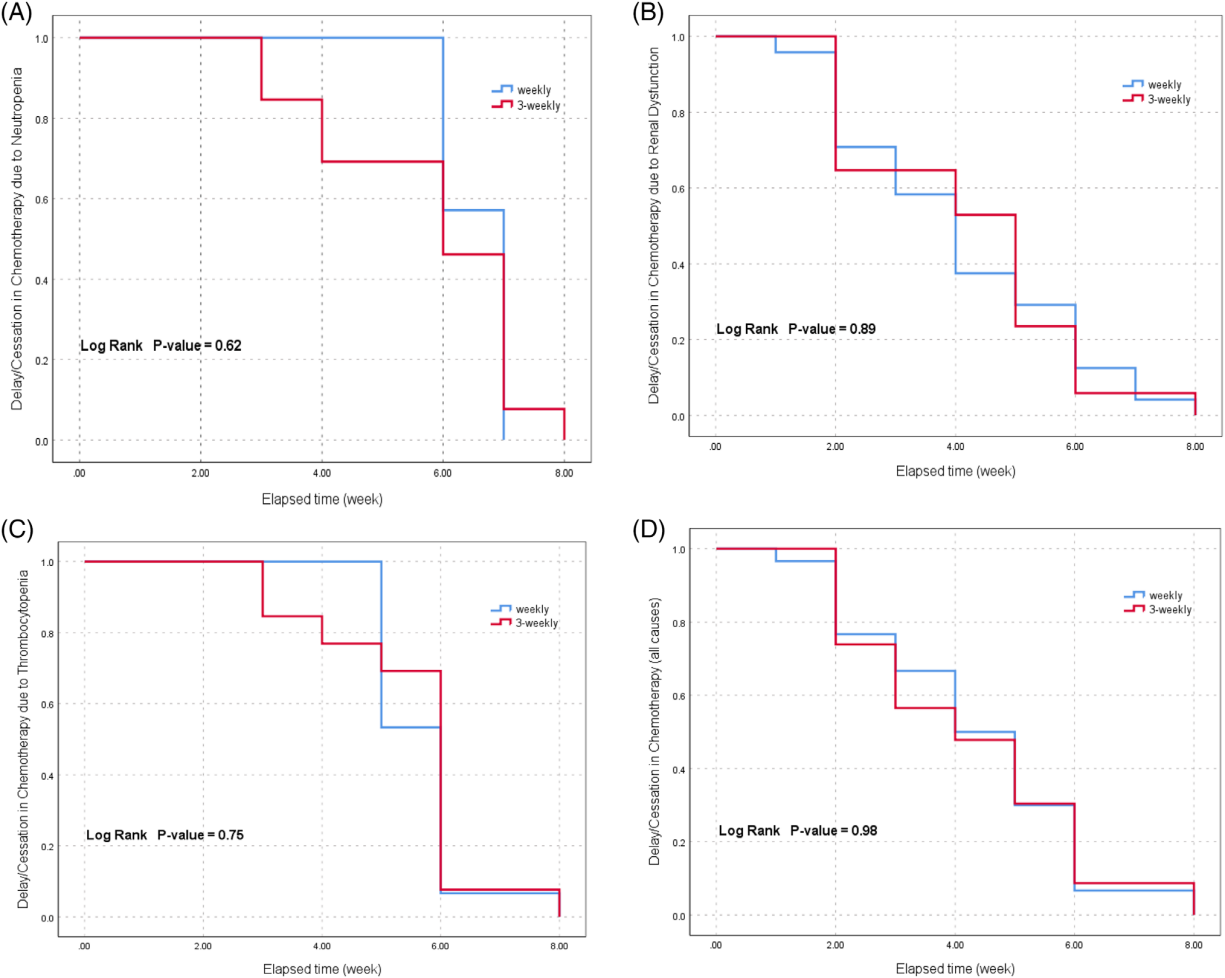
Kaplan–Meier curves of the time to occurrence of chemotherapy delay/cessation due to neutropenia (A), thrombocytopenia (B), renal dysfunction (C), and all three causes (D) in weekly and three‐weekly schedules

The median cycles of concurrent chemotherapy were 6.5 cycles in the weekly arm and 2.5 cycles in the three‐weekly arm. The number of patients who completed all planned chemotherapy was 16 (41%) and 15 (39.5%) for the weekly and three‐weekly groups, respectively. Dose adjustment was required in 33.3% and 42.1% of the participants in the weekly and three‐weekly groups, respectively, with no significant difference between arms (*P*‐value = .42). The leading cause of dose adjustment in the weekly group was thrombocytopenia (92.3%) while neutropenia was the most common cause (68.7%) in the three‐weekly group. Delay or discontinuation in the administration of chemotherapy was reported in 38.4% and 42.1% of the patients in the weekly and three‐weekly group, respectively (*P*‐value = .70). This was mainly due to renal dysfunction (73.3%) in the weekly arm and neutropenia (81.2%) in the three‐weekly arm (Table 2). The rates of neutropenia‐ and renal dysfunction‐induced delay/cessation in chemotherapy were significantly higher in three‐weekly and weekly arms, respectively (both *P*‐values = .001); however, the time to occurrence of adverse events was not different between the groups (Figure 2). The average duration and cumulative dose levels for renal disorder onset in the weekly group were 4.1 weeks and 247.5 mg/m^2^, and for neutropenia onset in the three‐weekly group were 5.76 weeks and 230 mg/m^2^, respectively.

Overall, 88.3% of the patients received ≥200 mg/m^2^ of cisplatin concurrent with radiotherapy. The number of cases that received ≥200 mg/m^2^ of cisplatin was greater in the weekly arm compared to the three‐weekly arm, although the difference was not significant statistically (*P*‐value = .08). Two participants in the weekly arm did not achieve the cut‐off due to development of renal dysfunction in one patients and severe paresthesia in another patient. In seven patients assigned to the three‐weekly group who received less than 200 mg/m^2^ cisplatin concurrent with radiotherapy, the leading cause of chemotherapy cessation was neutropenia (57.1%) accompanied with renal dysfunction and mucositis in three and two cases, respectively. The cumulative dose of cisplatin ≥240 mg/m^2^ was recorded in 66.2% of all patients. This rate was significantly greater in the weekly arm (79.5%) compared to the three‐weekly group (52.6%; *P*‐value = .01). In subgroup analysis, this difference was consistently significant in a definitive therapy setting (*P*‐value = .004).

The overall rate of grade 3–4 hematological toxicities was 61%. The overall rates of leukopenia, neutropenia, lymphopenia, anemia, and thrombocytopenia were 31.2%, 25.9%, 46.8%, 1.3%, and 5.2%, respectively. Compared with the participants assigned to the three‐weekly group, fewer grade 3–4 leukopenia and neutropenia were recorded in weekly schedule; the differences, however, were statistically nonsignificant (*P*‐value = .120 and .104, respectively). These findings were also true for the patients who received ≥240 mg/m^2^ of cisplatin concurrent with radiotherapy [26.1% vs. 46.6%, *P*‐value = .36 (for leukopenia), and 16.0% vs. 37.5%, *P*‐value = .23 (for neutropenia)]. Hematological toxicities are summarized in Table 3.

**TABLE 3:** Hematological toxicities

Hematologic toxicities grade ≤ 3	Weekly (*n* = 39)	Three‐weekly (*n* = 38)	*P*‐value
Leukopenia	23.1% (9)	39.5% (15)	.120
Neutropenia	17.9% (7)	34.2% (13)	.104
Lymphopenia	46.2% (18)	47.4% (18)	.915
Anemia	2.6% (1)	–	.999
Thrombocytopenia	5.1% (2)	5.3% (2)	.999
Overall hematologic toxicities	56.4% (22)	65.8% (25)	.399

The difference between weekly and three‐weekly regimens was nonsignificant in terms of either grade 3–4 creatinine rise (0.0% vs. 2.6%, *P*‐value = .32) or stage 3–4 AKI (2.6% vs. 2.5%, *P*‐value = .98). Generally, one patient experienced a marked Scr rise (who had received a three‐weekly schedule) and two patients, one in each group, developed stage 3 AKI. Among patients who received ≥240 mg/m^2^ of cisplatin concurrent with radiotherapy, the rates of grade 3–4 creatinine rise (0.0% vs. 4.5%, *P*‐value = .24) and stage 3–4 AKI (3.4% vs. 4.5%, *P*‐value = .84) were almost similar in weekly and three‐weekly regimens.

**FIGURE 3:**
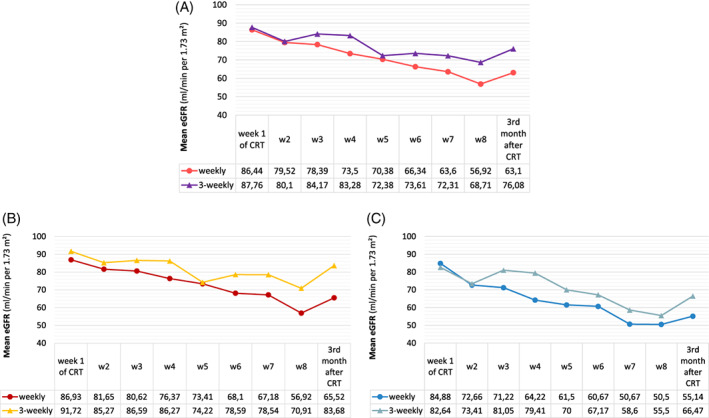
Estimated glomerular filtration rate for weekly and three‐weekly cisplatin during the chemoradiation (CRT) and 3 months after the treatment for all patients (A), patients who received ≥240 mg/m^2^ (B), and <240 mg/m^2^ of cisplatin concurrent with radiotherapy (C)

Figure 3 shows an overview of the eGFR between the weekly and three‐weekly groups. Although the mean eGFR level at the beginning of therapy was the same between the groups (86.44 and 87.76 mL/min/1.73 m^2^ for the weekly and three‐weekly schedules, respectively), a significant decrease of mean eGFR occurred over the course of treatment for both groups (Figure 3(A)). Based on the GEE analysis, the decrease in eGFR was more profound and statistically significant in the weekly arm compared to the three‐weekly arm (*P*‐value = .02). In the subanalysis, however, this difference was not observed in patients who received ≥240 mg/m^2^ of cisplatin concurrent with radiotherapy (*P*‐value = .11; Figure 3(B)).

## 4 DISCUSSION

Compelling evidence regarding toxicity profiles of various schedules of cisplatin administered in the form of chemoradiotherapy for LAHNC is lacking. Other agents (e.g., carboplatin, fluorouracil, cetuximab, or gemcitabine) may also be administered concurrently with radiotherapy.^17^, ^18^, ^19^ However, published literature supports the use of these agents as alternatives for patients who are not eligible for cisplatin.^17^ The current understanding of this topic is confined to retrospective and a few small prospective studies.^10^

In this trial, we aimed to compare the acute toxicity profile of the current standard chemoradiation regimen (i.e., three‐weekly cisplatin 100 mg/m^2^) with its alternative (i.e., weekly cisplatin 40 mg/m^2^). Each dosing schedule showed a different toxicity profile with increased renal impairment (as the main cause of delay in chemotherapy) and thrombocytopenia (as the main cause of dose modification) in a weekly regimen and more neutropenia (as the main cause of either delay in chemotherapy or dose modification) in the three‐weekly regimen. A comparative look at the GEE analyses of GFR, between all participants and those who received a higher cumulative dose of cisplatin concurrent with radiotherapy (i.e., ≥240 mg/m^2^), addresses the importance of the cumulative dose of cisplatin as a negative prognostic factor on renal function.

The current trial also demonstrated that patients in the two arms had similar risks of leukopenia, thrombocytopenia, and anemia, which is consistent with Guan et al.'s findings.^20^ However, Guan et al.'s meta‐analysis did not support our findings regarding delayed or interrupted chemotherapy. According to Guan's meta‐analysis, delay or interruption of chemotherapy was more common in weekly CCRT.^20^ In current study, the rate of delay/interruption of chemotherapy for weekly‐ and three‐weekly CCRT was 38.4% and 42.1%, respectively, and showing no statistically significant difference between groups. The main reasons for chemotherapy delay in weekly and three‐weekly CCRT arms were renal disorders and neutropenia, respectively. Uygun et al. reported chemotherapy interruption of 35% and 26% in weekly‐ and three‐weekly CCRT, respectively, without significant differences. The main reasons for the interruption were hematologic toxicity (85%) for the weekly group and both hematologic (50%) and renal toxicity (50%) for the three‐weekly group.^21^ As a result, our findings are in line with Uygun et al.'s study, although we found different reasons for a delay in chemotherapy.

In current trial, the rate of dose adjustment was 33% and 42% for weekly‐ and three‐weekly CCRT arms, respectively. The main reason for dose adjustment in either group was hematologic toxicity, which was thrombocytopenia (92.3%) for weekly and neutropenia (68.7%) for three‐weekly groups. In contrast, in Noronha et al.'s trial, the rate of dose reduction was much lower in that for weekly‐ and three‐weekly CCRT arms, the rates were 9.3% versus 8%, respectively, without significant difference. In their study, the underlying toxicity was not mentioned.^22^ Their results may be due to the utilization of a lower weekly dose of cisplatin (30 mg/m^2^). In Driessen et al.'s trial, the rate of renal toxicity was greater in the three‐weekly CCRT arm. In that trial, the rise in Scr was used as the criteria for renal toxicity.^23^ In our study, we used various criteria to evaluate the effect of cisplatin on renal functions, considering the uncertainty of the relation between Scr and eGFR.^24^ In this regard, the two criteria (i.e., Scr and AKI) were not significantly different across the two arms. However, the trend in eGFR reduction was significantly steeper in the weekly CCRT arm during the course of the trial (Figure 2). We administered magnesium sulfate just in three‐weekly CCRT arm while Driessen's group administered the substance in both arms. This may justify the discrepancy between the two studies. In addition, it highlights the importance of additional replacement of electrolytes in preventing renal dysfunction, as proposed by Faig et al.^25^ Moreover, in the current study, the cumulative dose of cisplatin was greater in the weekly schedule, which can justify the results.

According to Strojan et al.'s meta‐analysis, the efficacy of cisplatin concurrent with radiotherapy is dose‐dependent in that total doses of more than 200 mg/m^2^ are more efficacious compared to lower doses.^26^ Current trial showed a trend toward a more cumulative dose of cisplatin in the weekly schedule, which may, in turn, improve treatment outcomes. This finding is consistent with Bernier et al.'s study that alternative schedules of concurrent cisplatin (i.e., 30–40 mg/m^2^ weekly or 6 mg/m^2^ daily) improve compliance, which eventually results in a higher cumulative dose of cisplatin.^27^ While in current study, the number of patients who received cisplatin with a cumulative dose of ≥200 mg/m^2^ was similar, more patients in weekly group received a cumulative dose of ≥240 mg/m^2^ (*P*‐value = .01).

Several limitations of this clinical trial need to be acknowledged. First, during the trial, we encountered some unscheduled interruption of radiotherapy due to technical errors. This may have affected the toxicities, especially renal toxicities. Second, as is presented in Tables S1 and S2, the BC cancer guidelines suggest either “dose modification” or “treatment delay” options for three‐weekly CCRT (based on the renal function) while including only “delay in chemotherapy” option for weekly CCRT (based on the renal function). This specification may have biased the results of our trial regarding the difference in the effect of arms on renal function as we employed a “delay in treatment” (in case of creatinine clearance <50) only for weekly CCRT arm. This may have had some negative effects on the renal function of the weekly CCRT arm. Future trials with alternative dose adjustment criteria can address this issue. The third limitation of this study is related to the number of participants. The final sample comprised 15 cases less than the estimated number (eight cases for the experimental and seven cases for the control arms). Likely, this notion resulted in some statistically insignificant results. Therefore, future trials with more participants can reveal more accurate differences between the two arms. The fourth limitation of current study is that we restricted our evaluation only to renal and hematologic toxicities based on laboratory findings. We did not analyze other acute toxicities (e.g., mucositis). This may have affected the results. Considering other issues in future trials, including other toxicities and the measure of the quality of life, among others, could provide more reliable results.

## 5 CONCLUSIONS

The results showed that the weekly schedule of concurrent cisplatin has its pros and cons in comparison with the three‐weekly arm, which should be considered in clinical practice. Its advantages over the three‐weekly regimen include a larger cumulative dose and less chemotherapy delay/interruption because of neutropenia. However, its important drawback is related to renal impairment. From a methodological perspective, the study emphasizes the necessity of ad hoc randomized clinical trials with larger sample sizes to validate these findings.

## CONFLICT OF INTEREST

The authors have no relevant relationships to disclose.

## AUTHOR CONTRIBUTIONS

All authors had full access to the data in the study and take responsibility for the integrity of the data and the accuracy of the data analysis. Conceptualization, A.A. and S.N.; Methodology, A.A., S.N., and F.T.H.; Investigation, S.N. and F.T.H.; Formal Analysis, F.T.H.; Resources, A.A., A.S., S.A., and K.N.; Writing ‐ Original Draft, F.T.H; Writing ‐ Review & Editing, A.A. and F.T.H.; Visualization, K.N.; Supervision, A.A.; Funding Acquisition, None.

## ETHICAL STATEMENT

Before commencing the study, the protocol of the study was approved by Ethics Committee of Shahid Beheshti University of Medical Sciences. The study was registered in the Iranian Registry Clinical Trial on March 12, 2018 (Registration number: IRCT20180223038829N1). Before the assignment of patients to treatment groups, written informed consent and their consent to publish their data were obtained from the patients.

## Supporting information


**Supplementary Table S1** Dose modification based on creatinine clearance (according to BC cancer guidelines)
**Supplementary Table S2**. Dose modification based on hematologic toxicities (according to BC cancer guidelines)

## Data Availability

Data are available upon request.
