# Supplementary material for: Randomized trial on acute toxicities of weekly vs three‐weekly cisplatin‐based chemoradiation in head and neck cancer
Source: Cancer Rep (Hoboken). 2021 Jun 7;5(1):e1425. doi: 10.1002/cnr2.1425 (PMC8789619; doi:10.1002/cnr2.1425)
Supplement: Supplementary file 1 — Supplementary Table S1 Dose modification based on creatinine clearance (according to BC cancer guidelines) Supplementary Table S2. Dose modification based on hematologic toxicities (according to BC cancer guidelines) [file CNR2-5-e1425-s001.docx]

Supplementary table 1. Dose modification based on creatinine clearance (according to BC cancer guidelines)

| Dosing schedule | Creatinine clearance (ml/min) | Dose |
| --- | --- | --- |
| Low dose cisplatin | Less than 50 | Delay chemotherapy, recheck in 1 week |
|  | Greater than or equal to 50 | Continue chemotherapy |
| High dose cisplatin | Less than 45 | Hold or delay chemotherapy |
|  | 45-59 | 80% cisplatin |
|  | Greater than or equal to 60 | 100% cisplatin |

Supplementary table 2. Dose modification based on hematologic toxicities (according to BC cancer guidelines)

| Dosing schedule | ANC (×10^3^ ml^-1^) |  | Platelet (×10^3^ ml^-1^) | Dose |
| --- | --- | --- | --- | --- |
| Low dose cisplatin | Less than 0.8 | or | Less than 100 | 50% cisplatin |
|  | Greater than or equal to 0.8 | and | Greater than or equal to 100 | 100% cisplatin |
| High dose cisplatin | Less than 1 | or | Less than 75 | Delay one week |
|  | 1-1.49 | or | 75-99 | 75% cisplatin |
|  | Greater than or equal to 1.5 | and | Greater than or equal to 100 | 100% cisplatin |

ANC, Absolute neutrophil count
